# ChatHTN: a consultation model for hypertension

**DOI:** 10.1038/s41598-026-47937-1

**Published:** 2026-04-09

**Authors:** Dongmei Wang, Zhongxu Yuan, Hua Deng, Zhangjun Peng

**Affiliations:** 1 Mianyang Fulin Hospital, Mianyang, 621000 China; 2 School of Computer Science and Technology, Southwest University of Science and Technology, Mianyang, 621010 China; 3 Sichuan College of Traditional Chinese Medicine, Mianyang, 621010 China

**Keywords:** Hypertension, Large language models, Knowledge graph, Chain of thought, Computational biology and bioinformatics, Health care, Mathematics and computing

## Abstract

The rapid development of large language models (LLMs) has greatly advanced natural language processing (NLP). While these models perform remarkably well in tasks such as text generation and translation, they still face challenges in highly specialized domains such as hypertension, where domain expertise and personalization are crucial. To address these limitations, we introduce a generative framework for intelligent hypertension consultation that combines a domain-specific knowledge graph, a multi-task fine-tuning strategy, and a specialized dataset. The knowledge graph enhances the model’s medical knowledge, while the multi-task fine-tuning mechanism optimizes tasks like medical entity recognition and etiology classification to ensure consistency. To further strengthen reasoning ability, we construct HTN-5M, a large-scale Chinese dataset that embeds chain-of-thought (CoT) reasoning in a structured triplet format (input, output, CoT), supporting both question-answer generation and auxiliary learning. Experimental results demonstrate that our approach outperforms strong baselines, achieving a 16.25% improvement over DeepSeek-LLM-67B-base on the CMB benchmark and an approximate 10% gain over HuatuoGPT across three medical datasets.

## Introduction

Hypertension affects over 1.3 billion people globally^1^, often asymptomatic in its early stages. Poor long-term control can lead to serious complications, including stroke, heart failure, and renal impairment^2,3^. Despite its status as the leading risk factor for premature death, hypertension remains poorly controlled, particularly in developing countries^4^. In China, the prevalence of hypertension among adults has reached nearly 30 percent, yet awareness, treatment, and control rates remain far below optimal levels^5^. Given the coexistence of high prevalence and low control, developing intelligent consultation systems with strong knowledge understanding and reasoning capabilities has become an important direction for improving chronic disease management. The advent of the Transformer architecture has accelerated the development of large language models (LLMs), which excel in natural language understanding and generation. However, when applied to highly specialized domains such as medicine and law, LLMs frequently produce “hallucinations,”generating content without factual grounding or providing irrelevant answers^6–8^.

Many researchers have addressed these challenges by adopting domain-specific fine-tuning of general-purpose models, such as BianQue^9^, which improved performance in Chinese medical dialogue tasks. ChatGLM-Med^10^, built upon the ChatGLM^11^ architecture, incorporates multi-source medical knowledge and literature through instruction tuning, significantly enhancing its performance in medical question answering, clinical reasoning, and health consultation scenarios. Similarly, in the legal domain, models such as LEGAL-BERT^12^ and Lawformer^13^ have been fine-tuned on legal judgments, statutes, and other domain-specific corpora, achieving notable gains in legal text understanding, clause matching, and judicial question answering. These studies further validate the feasibility and effectiveness of parameter-efficient tuning methods such as LoRA^14^ and P-Tuning^15^ for enhancing model performance in specialized domains. In the medical context, however, the reliability, accuracy, and interpretability of generated outputs are especially critical, as model recommendations can directly influence clinical decisions, medication choices, and even patient safety. Prior research^16,17^ has highlighted that language models without professional supervision or external knowledge grounding are prone to generating misleading medical advice, which in severe cases may result in misdiagnosis or inappropriate treatment.

To solve the above problems and to enhance the professionalism and reliability of models in hypertension question answering, We propose a generative model that combines a hypertension knowledge graph with a multi-task fine-tuning framework, improving the model’s ability to understand and generate medical terms, causal mechanisms, and personalized interventions. Based on authoritative medical literature, hypertension prevention and treatment guidelines, and clinical consultation data, we construct a hypertension knowledge graph that covers six core domains such as etiologies and clinical symptoms. The graph is organized in the form of entity–relation–entity triples, where the entities include six categories such as complications and interventions, and the relations include four types such as common medications and treatment methods. After construction, medical knowledge is incorporated into the representation space of the generative model through TransE-based knowledge-graph embeddings^18^, which enhance the model’s medical knowledge coverage during the reasoning process. In designing the multi-task fine-tuning framework, we divide key information extraction tasks in hypertension consultation into four subtasks: medical entity recognition, etiology classification, complication identification, and intervention suggestion generation. The medical entity recognition task improves the model’s ability to identify terms such as“elevated blood pressure”and“diuretics.” Etiology classification distinguishes between primary and secondary hypertension. complication identification focuses on modeling the associations between hypertension and diseases such as stroke and renal impairment. Intervention suggestion generation provides personalized lifestyle recommendations such as salt restriction, exercise, and weight loss based on patient characteristics. Each subtask is optimized with an independent cross-entropy loss function and trained collaboratively with the main task of hypertension question answering. Built on shared representations, this design jointly drives model learning and significantly improves the accuracy and medical consistency of the generated results.

The main contributions of this paper are as follows:

- This study introduces a generative model that integrates a hypertension-specific knowledge graph with a multi-task fine-tuning mechanism to enhance the professionalism and personalization of hypertension consultations. By incorporating structured medical knowledge and auxiliary tasks such as medical entity recognition and etiology classification, the model strengthens its ability to understand key factors including causes, symptoms, and interventions. Compared with existing models such as HuatuoGPT, it achieves 73% accuracy in hypertension consultations, with improved precision in medical terminology and consistency in clinical reasoning.
- In addition, we construct HTN-5M, a Chinese question–answer dataset for hypertension that embeds CoT reasoning. Organized as triplets of clinical questions, answers, and reasoning paths, it enables the model to mimic physicians’ reasoning processes and generate more interpretable and logically coherent responses.
- Extensive evaluations on multiple medical benchmarks confirm that our approach outperforms both general-purpose and domain-adapted models, particularly in tasks requiring multi-step reasoning and complex etiology matching. These results highlight the effectiveness of combining knowledge-graph augmentation with multi-task learning for intelligent hypertension consultation.

## Related work

### Large language models in the medical domain

The rapid evolution of Large Language Models (LLMs) has catalyzed significant advancements in intelligent healthcare. While general-purpose models like ChatGPT^19^ and Gemini^20^ demonstrate impressive natural language understanding, they often lack the domain-specific depth required for clinical applications. To address this, researchers have focused on adapting general LLMs to the medical domain through instruction tuning and continuous pre-training. Notable examples include DoctorGLM^21^, which fine-tuned ChatGLM-6B on Chinese medical dialogues to enhance professional responsiveness. Similarly, HuatuoGPT^22,23^integrated real-world medical data with distilled knowledge to improve diagnostic accuracy and complex reasoning capabilities. BenTsao^24^ further explored parameter efficient fine-tuning (PEFT) methods like LoRA to construct lightweight medical models. Despite these achievements, most existing medical LLMs rely heavily on unstructured dialogue data. In highly specialized domains like hypertension management, the lack of structured constraints often leads to “hallucinations”[6]-generating plausible but clinically incorrect advice-highlighting the need for models that can strictly adhere to medical guidelines and evidence-based practices.

### Knowledge-enhanced medical reasoning

To mitigate the limitations of pure data-driven generation, integrating structured knowledge into LLMs has become a critical research direction. Knowledge Graphs (KGs) provide a factual grounding that is essential for medical reliability. Previous works have explored various integration strategies. For instance, GreaseLM^25^ and KG-CoT^26^ demonstrated that fusing KG representations with language models significantly enhances reasoning in multi-hop question answering. In the medical field, methods like K-BERT^27^ inject domain knowledge (e.g. drug-disease interactions) directly into the input sequence to guide the model’s inference. Other approaches, such as Sobolev et al. (Note: or generic description if ref not available), leverage retrieval-augmented generation (RAG) to fetch relevant guidelines before generating responses. However, existing knowledge-enhanced methods often focus on simple entity matching or retrieval, which may be insufficient for the complex, chain-of-thought reasoning required in chronic disease consultation. Our work extends these methodologies by embedding a hypertension-specific knowledge graph directly into the LLM’s semantic space and coupling it with a multi-task objective to ensure that generated advice is not only factually accurate but also logically consistent with clinical reasoning paths.

### Hypertension question answering model

The application of LLM in healthcare has drawn growing interest, particularly in hypertension-related question answering. Kusunose et al^28^. evaluated ChatGPT on the Japanese Society of Hypertension Guidelines (2019), reporting 80% accuracy in clinical scenario questions but only 36% in guideline-based queries, underscoring its limitations with professional details and authoritative knowledge. To address such gaps, Wu et al^29^. developed the“Qingting”LLM on Baichuan2 with full fine-tuning, tool-use mechanisms, and uncertain knowledge graph–based retrieval augmentation, improving performance in chronic disease QA including hypertension. Liu et al^30^. further designed a system combining a hypertension knowledge graph with intent recognition and NER, which interprets queries accurately, generates professional responses, and guides users toward clearer questions, enhancing reliability in clinical consultations. These works show that knowledge graphs and domain-specific tuning can improve LLM performance in hypertension QA, yet issues such as incomplete knowledge coverage and limited clinical adaptability remain. Beyond hypertension, recent work such as AMIE^31^ has demonstrated that conversational diagnostic AI systems based on LLMs can approach or even surpass primary-care physicians in OSCE-style evaluations under a supervised, physician-in-the-loop workflow, highlighting both the potential and the safety requirements of deploying such systems in real clinical settings. To overcome these challenges in the specific context of hypertension, this study builds a hypertension knowledge graph and introduces a multi-task fine-tuning framework to enhance medical reasoning and accuracy, providing a foundation for more trustworthy hypertension consultation systems.

## Method

**Fig. 1:**
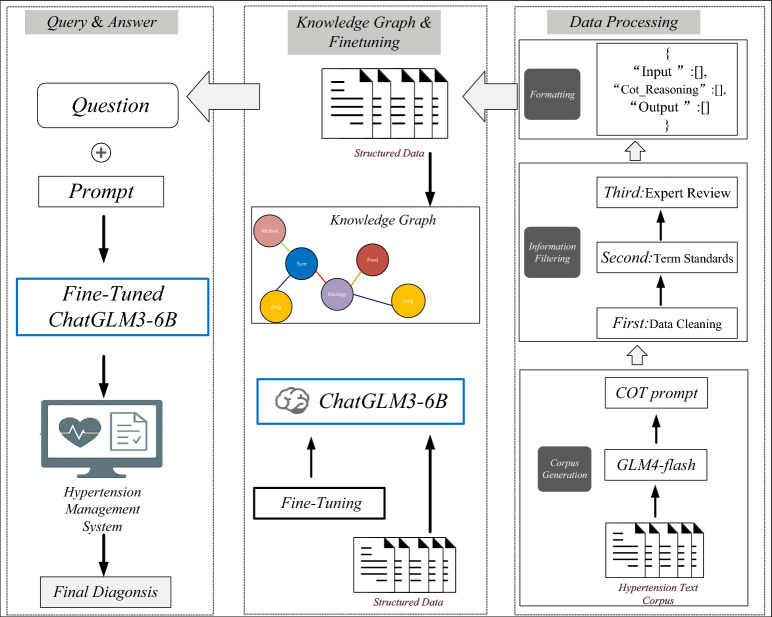
Overview of ChatHTN model.

As shown in Fig. 1, the proposed framework consists of three core modules: data processing, knowledge-graph–based model training, and intelligent consultation system construction. First, through automatic data cleaning and medical terminology standardization, we build a high-quality hypertension consultation dataset that provides a reliable foundation for model training. Second, a hypertension knowledge graph is constructed from authoritative medical resources to serve as external knowledge for generation. The graph is integrated into the model via TransE-based knowledge-graph embeddings and combined with a multi-task fine-tuning mechanism, where the main task is jointly trained with multiple auxiliary tasks to enhance semantic understanding and ensure consistency in question–answer generation. Finally, an intelligent hypertension consultation system is developed on top of the trained model, enabling efficient and professional interactive consultations.

**Table 1:** HTN-5M dataset.

Entity type	Number
Symptoms	3437
Drugs	6218
Treatments	4682

### Dataset construction

**Fig. 2:**
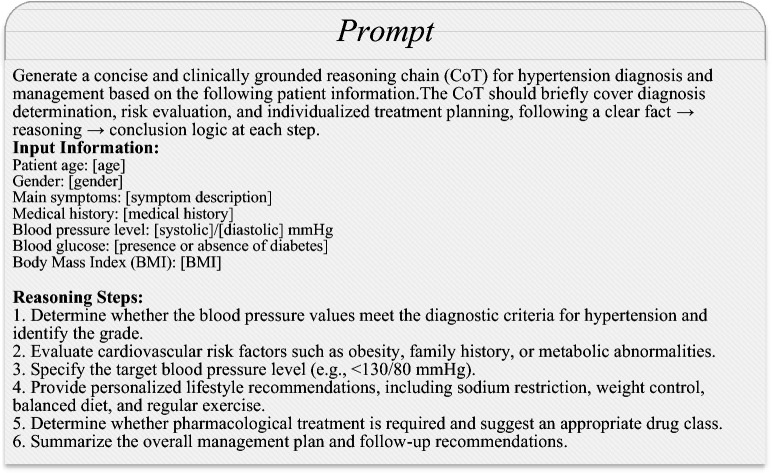
Prompt template for generating COT.

As shown in Table 1, the HTN-5M dataset was generated through a combination of model-based generation and expert curation, ensuring medical reliability via multi-stage filtering and validation. The construction process consists of three main stages. (1) Corpus generation: Using the GLM4-Flash API, prompts related to hypertension – including key information such as chief complaints, medical history, and lifestyle habits – are provided to automatically generate complete question–answer pairs with chain-of-thought reasoning. The model first produces reasoning text aligned with medical logic and then generates structured and coherent clinical responses; the prompt template for CoT generation is shown in Fig. 2. GLM4-Flash is used only to bootstrap a large draft corpus with consistent structure and high generation throughput, which enables efficient coverage of diverse hypertension scenarios. Importantly, the generated samples are not treated as final supervision: they must pass the subsequent three-level filtering pipeline and senior-physician validation described in Stage (2). Therefore, the final HTN-5M corpus reflects expert-validated clinical quality rather than the raw behavior of the generator. (2) Information filtering and medical validation: A three-level pipeline is applied to improve accuracy and reduce noise. First, regular expressions are used for automatic cleaning, including removing abnormal symbols, unifying units, and eliminating redundant or duplicate content. Second, a medical terminology lexicon is applied to standardize key clinical terms and ensure consistency. Third, senior physicians perform expert review of the remaining samples, checking the logical validity of the reasoning chains, the compliance of diagnostic and treatment recommendations with clinical guidelines, and the rigor of language; samples that fail this check are revised or discarded. (3) Structured output: All validated samples are converted into a nested JSON format for subsequent model training and system integration. Each entry contains three core fields: input, recording the original patient question or symptom description (e.g. “I have been feeling dizzy, and my blood pressure is 160/95. Do I need to take medication?”); cot_reasoning, recording the model-generated step-by-step reasoning that mimics physicians’ diagnostic, causal, and therapeutic decision-making processes; and answer, recording the final professional medical advice, expressed clearly and with actionable guidance. To mitigate potential homogeneity in LLM-bootstrapped data, we explicitly remove redundant or near-duplicate samples during automatic cleaning and require senior physicians to flag template-like repetitions in addition to correctness checks. Moreover, in training we do not rely on HTN-5M alone; instead, we combine HTN-5M with additional corpora to expose the model to more diverse clinical expressions.

To ensure its medical reliability, we applied a thorough filtering and expert review process. (1) Expert Review and Medical Validation: A total of 5 senior medical professionals reviewed the 3,437 samples, ensuring that diagnostic reasoning and treatment recommendations adhered to clinical standards. 98.5% of the samples passed the review, with a small percentage flagged for refinement, confirming the medical soundness of the data. (2) Bias Mitigation: A multi-step process was applied to reduce biases. First,Data Cleaning: Removal of irrelevant symbols and redundant content led to a 3% reduction in noise. second,Terminology Standardization: A medical lexicon reduced term variability by 12%, ensuring consistent clinical terminology. Third, Logical Validation: We identified and removed 5% of invalid entries, improving logical consistency and medical relevance.

While HTN-5M primarily targets common hypertension consultation scenarios, we also include samples with secondary-hypertension indicators and complication/target-organ-damage descriptions, consistent with our auxiliary supervision (etiology classification and complication identification). We acknowledge that truly rare or institution-specific presentations may still be underrepresented in an LLM-bootstrapped corpus; therefore, we treat HTN-5M as a clinically validated foundation and plan to further enrich long-tail coverage with real-world retrospective consultation records in future work.

**Fig. 3:**
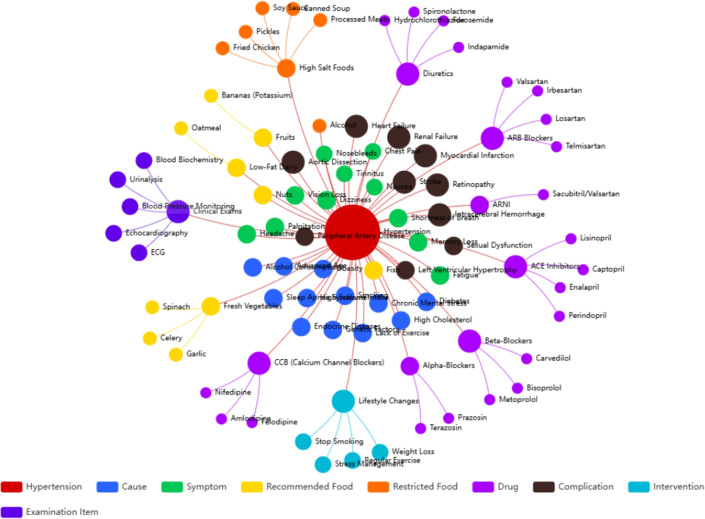
An example of the knowledge graph.

### Construction and embedding of knowledge graphs

To ensure the professionalism and traceability of the medical knowledge, we constructed a high-quality Hypertension Knowledge Graph (HTN-KG) through a rigorous pipeline. As shown in Table  2, the graph covers 6 core entity types (e.g. Drugs, Symptoms, Foods) and 4 major relation types, derived from the “Guidelines for the Prevention and Treatment of Hypertension in China” and authoritative clinical data. Construction Pipeline and Statistics: The construction process involved three key stages. (1) Data Acquisition: We curated a corpus from authoritative sources, including clinical guidelines, research articles, and physician consultation records. (2) Information Extraction: To extract structured triples, we employed a pipeline approach. A fine-tuned language model was applied to extract medical entities and identify semantic links from unstructured text. (3) Knowledge Fusion and Validation: Extracted entities underwent disambiguation and alignment. To ensure clinical safety, senior physicians reviewed the sampled triples to correct conflicting facts. The final HTN-KG contains approximately 33452 entities and 147210 relations. The detailed distribution is presented in Table 2 . The graph exhibits a scale-free characteristic, with “Hypertension” and common drugs serving as dense hub nodes. The sample diagram is shown in the Fig. 3.

**Table 2:** Detailed statistics and distribution of the hypertension knowledge graph (HTN-KG).

Category	Type	Count	Description/examples
**Entities**	Symptoms	8420	Dizziness, Palpitation, Edema
	Drugs	6218	CCB, ARB, Diuretics, Beta-blockers
	Foods	5100	Celery, Sodium-rich foods, Low-fat dairy
	Complications	3850	Stroke, Renal failure, Retinopathy
	Interventions	4682	Aerobic exercise, Salt restriction
	Etiologies	5182	Genetic factors, Sleep apnea
	**Total Entities**	**33452**	–
**Relations**	Treat	45,200	Drug$\xrightarrow {\text {treats}}$Symptom
	Cause	32,150	Etiology$\xrightarrow {\text {causes}}$Hypertension
	Has_Side_Effect	28,400	Drug$\xrightarrow {\text {has}}$Symptom
	Recommend/Avoid	41,460	Hypertension$\xrightarrow {\text {avoid}}$Food
	Total relations	147210	–

*Knowledge graph embedding strategy:* After construction, medical knowledge is incorporated into the generative model via embeddings. We selected TransE for this purpose based on a strategic trade-off between computational efficiency and representation sufficiency5. We acknowledge that models like TransR are more expressive in handling 1-to-N relationships but come with significantly higher parameter complexity ($O(d \times k)$ versus *O*(*d*) for TransE).

However, to address the dimensionality mismatch and integrate these structural priors into the LLM architecture, we designed a projection-based injection mechanism. Specifically, for a retrieved knowledge triple (*h*, *r*, *t*), we obtain its embedding vector ${\bf v}_{trip} = {\bf v}_h + {\bf v}_r + {\bf v}_t$, where ${\bf v} \in \mathbb {R}^{d_{kg}}$. Since the dimension of the knowledge graph embeddings ($d_{kg}$) differs from the hidden dimension of ChatGLM3-6B ($d_{llm}$), we introduce a learnable linear projection matrix ${\bf W}_{proj} \in \mathbb {R}^{d_{llm} \times d_{kg}}$. The projected knowledge embedding ${\bf E}_{kg}$ is calculated as

1 $$\begin{aligned} {\bf E}_{kg} = {\bf W}_{proj} \cdot {\bf v}_{trip} \end{aligned}.$$

Finally, ${\bf E}_{kg}$ is concatenated to the front of the text embedding sequence as a continuous “soft prompt.” This allows the self-attention mechanism of the LLM to directly attend to the structured medical knowledge alongside the textual context, effectively compensating for the potential semantic limitations of TransE while grounding the generation in authoritative guidelines.

### Multi-task knowledge fine-tuning strategy

To better adapt the ChatGLM3-6B model for hypertension consultations, we designed a multi-task fine-tuning framework tailored to intelligent question answering in this domain. Within this framework, the key information extraction tasks required for hypertension consultations are divided into four interrelated subtasks: medical entity recognition, etiology classification, complication identification, and intervention suggestion generation, together with the final hypertension question answering generation task. Each subtask is associated with a specific loss function, and the overall training objective is a weighted combination of these individual losses. we first define the loss for each subtask and then describe the joint multi-task objective and the strategy used to select an appropriate set of loss weights.

*Hypertension medical entity recognition (H-MER).* This task requires the model to accurately identify medical terms in text that are relevant to hypertension diagnosis and treatment, and classify them into predefined entity categories. For example, in the sentence “The patient reported a significant increase in blood pressure, which was relieved after taking diuretics”, the model should recognize “increase in blood pressure”as a Symptom and “diuretics”as a Drug. Formally, given an input word sequence $X = \{x_{1}, x_{2}, \ldots , x_{T}\}$, where *T* denotes the sequence length, the model is required to predict an entity label $y_{t} \in \mathcal {Y}$ for each token $x_{t}$. Here, $\mathcal {Y}$ represents the predefined label set, for example: B-Symptom (beginning of a symptom), I-Symptom (inside a symptom), B-Drug (beginning of a drug), and O (outside any entity).

2 $$\begin{aligned} \mathcal {L}_{\text {H-MER}} = -\frac{1}{T} \sum _{t=1}^{T} \sum _{c \in \mathcal {Y}} y_t^{(c)} \log \left( p_t^{(c)}\right) , \end{aligned}$$

where $y_t^{(c)}$ represents the one-hot encoding of token $x_t$ with respect to the true label category *c* (the value is 1 if the true label is *c*, and 0 otherwise), and $p_t^{(c)}$ denotes the predicted probability that token $x_t$ belongs to category *c*.

*Hypertension etiology classification (H-EC).* This task involves automatically classifying hypertension as either Essential Hypertension or Secondary Hypertension based on patient descriptions. For example, if the description mentions “adrenal adenoma causing increased blood pressure”, it should be classified as “Secondary Hypertension”. The model predicts the probability distribution of the etiology category $\hat{y} \in \mathbb {R}^{K}$ from the input text *X*, where *K* is the number of categories (usually $K = 2$, corresponding to Essential and Secondary Hypertension):

3 $$\begin{aligned} \mathcal {L}_{\text {H-EC}} = -\sum _{k=1}^{K} y^{(k)} \log \left( \hat{y}^{(k)} \right) , \end{aligned}$$

where $y^{(k)}$ is the one-hot encoding vector of the true etiology category, and $\hat{y}^{(k)}$ is the probability predicted by the model that the sample belongs to the *k*-th category.

*Hypertension complication identification (H-CI).* This task is primarily used to identify complications or target organ damage clearly associated with hypertension mentioned in the text. For example, from the clinical description “Hypertension history for 10 years, recent onset of renal impairment”, the model needs to accurately identify “renal impairment” (labeled as *Renal*_*Impairment*). This task is modeled as a multi-label classification problem because patients may have multiple complications simultaneously or sequentially. The model needs to predict a probability vector of complications, $\hat{z} \in \mathbb {R}^{M}$, where *M* is the total number of predefined complication categories. Typical categories include: *Stroke*, *Myocardial*_*Infarction*, *Renal*_*Impairment*, etc.

4 $$\begin{aligned} \mathcal {L}_{\text {H-CI}} = -\frac{1}{M} \sum _{m=1}^{M} \left[ z^{(m)} \log \hat{z}^{(m)} + \bigl (1 - z^{(m)}\bigr ) \log \bigl (1 - \hat{z}^{(m)}\bigr ) \right] , \end{aligned}$$

where $z^{(m)} \in \{0, 1\}$ is the true label indicating whether the *m*-th complication is mentioned in the text (1 if present, 0 otherwise), and $\hat{z}^{(m)}$ is the predicted probability that the *m*-th complication is present.

*Hypertension intervention suggestion generation (H-ISG).* Based on the patient’s specific characteristics (such as age, symptoms, blood pressure level, complications, and mentioned lifestyle factors), this task generates a personalized sequence of non-pharmacological health recommendations $S = \{s_{1}, s_{2}, \ldots , s_{U}\}$, where *U* is the length of the suggestion sequence. For example, for a patient with obesity and a high-salt diet, the generated recommendation could be: “Limit daily salt intake to under 5 g, engage in at least 150 minutes of moderate-intensity aerobic exercise weekly, and gradually reduce weight to achieve a $\textrm{BMI} < 24$.”

In addition, the knowledge graph includes “food”as an entity type, and dietary recommendations have been integrated into the H-ISG subtask. During modeling, food-related entities are categorized according to their effects on blood pressure regulation, for instance, foods recommended for hypertensive patients (e.g. potassium-rich fruits, vegetables, whole grains) and foods that should be limited (e.g. high-sodium or high-fat foods). These entities are embedded into the model’s representation space together with other lifestyle factors. When generating intervention suggestions, the model retrieves relevant food entities from the knowledge graph based on patient characteristics and blood pressure level, thereby producing individualized dietary recommendations consistent with hypertension management guidelines.

5 $$\begin{aligned} \mathcal {L}_{\text {H-ISG}} = -\sum _{u=1}^{U} \log P(s_u \mid s_{<u}, X), \end{aligned}$$

where $P(s_u \mid s_{<u}, X)$ represents the probability that the model predicts the correct next suggestion token $s_u$, given the input text *X* and the previously generated suggestion prefix $s_{<u}$.

*Hypertension question answering generation (H-QAG).* This task involves generating an accurate, fluent, and medically consistent answer text sequence $A = \{a_{1}, a_{2}, \ldots , a_{V}\}$ based on the patient’s consultation description *X* and a given question *Q*:

6 $$\begin{aligned} \mathcal {L}_{\text {H-QAG}} = -\sum _{v=1}^{V} \log P(a_v \mid a_{<v}, Q, X), \end{aligned}$$

where *V* represents the length of the generated answer text, and $a_{<v}$ refers to the first $v-1$ tokens of the sequence already generated (the prefix sequence).

These subtasks are interrelated and mutually reinforcing through shared underlying representations: medical entity recognition enhances the model’s understanding of key terms, etiology and complication classification provides crucial diagnostic context, and intervention suggestion generation strengthens the model’s ability to capture health management knowledge. Together, these pieces of information provide a richer, more structured, and more accurate medical context representation for the final hypertension question answering generation (H-QAG) task, driving the model to generate answers with higher accuracy and stronger medical consistency.

*Joint multi-task objective and loss weight selection.* To jointly optimize all subtasks, we combine the above loss functions into a single multi-task objective:

7 $$\begin{aligned} \mathcal {L}_{\text {total}} = \lambda _{\text {MER}} \mathcal {L}_{\text {H-MER}} + \lambda _{\text {EC}} \mathcal {L}_{\text {H-EC}} + \lambda _{\text {CI}} \mathcal {L}_{\text {H-CI}} + \lambda _{\text {ISG}} \mathcal {L}_{\text {H-ISG}} + \lambda _{\text {QAG}} \mathcal {L}_{\text {H-QAG}}, \end{aligned}$$

where $\lambda _{\text {MER}}$, $\lambda _{\text {EC}}$, $\lambda _{\text {CI}}$, $\lambda _{\text {ISG}}$ and $\lambda _{\text {QAG}}$ control the relative contributions of each subtask.

In practice, H-ISG and H-QAG are the main clinical objectives, while H-MER, H-EC and H-CI provide complementary supervision that improves the model’s understanding of patient status and disease progression. Therefore, we assign slightly larger weights to H-ISG and H-QAG and comparable but smaller weights to the auxiliary subtasks. Concretely, we first perform a coarse grid search over candidate weight combinations, restricting each $\lambda$ to the set $\{0.5, 0.8, 1.0, 1.2\}$ and ensuring that $\lambda _{\text {ISG}}, \lambda _{\text {QAG}} \ge \lambda _{\text {MER}}, \lambda _{\text {EC}}, \lambda _{\text {CI}}$. For each combination, we train the model on the training set and evaluate BLEU-4, ROUGE-L, METEOR and CMB on a held-out validation set.

Representative results of this sensitivity analysis are reported in Table 6 (Sect. "Performance across question difficulty levels (RQ1–RQ3)"). Based on these experiments and feedback from two hypertension specialists, we select the configuration $\lambda _{\text {MER}} = 0.8$, $\lambda _{\text {EC}} = 0.6$, $\lambda _{\text {CI}} = 0.6$, $\lambda _{\text {ISG}} = 1.0$ and $\lambda _{\text {QAG}} = 1.0$ for all subsequent experiments. This setting achieves the best overall validation performance while maintaining a good balance between entity-level accuracy, etiology and complication identification, and the quality of generated intervention suggestions and final answers.

**Fig. 4:**
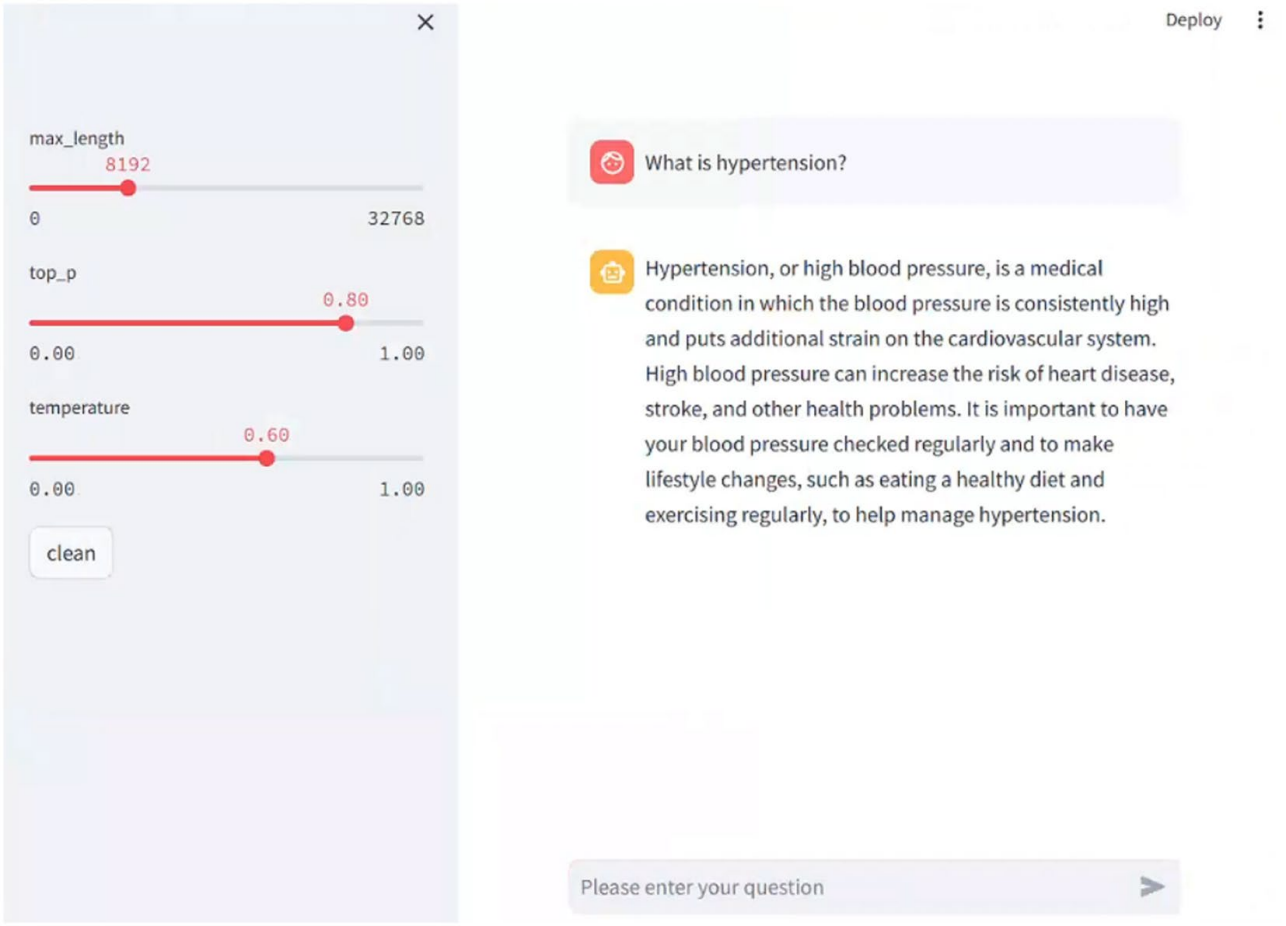
Example diagram of a dialogue system.

### Inquiry and diagnosis system

To facilitate efficient access to the intelligent consultation model for end users (including patients and doctors), this study developed a web-based hypertension consultation system using the Gradio framework, as shown in the Fig. 4. The system is deployed on a local server and enables natural language interaction through a browser interface, offering good responsiveness and privacy protection. The Gradio framework allows for the rapid construction of a front-end system with a conversational interface, improving the interaction efficiency between the model and the users.Upon system startup, the fine-tuned model weights are first loaded and initialized as a callable inference engine. If the hardware environment supports it, GPU acceleration is automatically enabled to speed up inference. Next, the core dialogue function is defined as the model’s inference interface, responsible for receiving user questions, processing contextual information, and generating personalized professional responses. The system also supports multi-turn dialogue tracking, allowing it to continuously integrate users’ multi-turn consultation information and dynamically update medical recommendations, further enhancing user experience.

To ensure user data security and compliance, the entire system can be run on a local server, ensuring that users’ health data is not uploaded to the cloud or third-party platforms, meeting the strict privacy protection requirements for sensitive health information in the medical context. Additionally, this hypertension intelligent consultation platform is flexible in deployment and privacy-friendly, allowing it to serve as an online health consultation tool for patients or be deployed within healthcare institutions to assist doctor-patient communication and chronic disease management. These features highlight the broad adaptability and practical implementation potential of large language models in hypertension and other chronic disease management domains.

## Experiment

### Composition of the dataset

This study constructs a structured Chinese hypertension consultation dataset to train and evaluate intelligent consultation systems. All samples from different sources are first mapped to a unified three-field schema: patient input (input), the model’s step-by-step reasoning (cot_reasoning), and the final answer (answer), ensuring that chain-of-thought reasoning is explicitly represented before generating diagnostic suggestions. To enhance medical knowledge coverage while avoiding over-reliance on any single corpus, we integrate several open-source medical datasets. Table 3 summarizes the number of samples and train–test splits for each dataset, and the detailed descriptions are given below.

**Table 3:** Summary statistics of the datasets used in this study.

Dataset	Training samples	Test samples	Share of training set (%)
HTN-5M	2750	687	36.4
MedDG	4000	1000	53.0
MIMIC-III	800	200	10.6
CMB	–	500	–
Total	7550	2387	100.0

HTN-5M: An LLM-assisted bootstrapped dataset where GLM4-Flash is used to generate initial draft consultations under structured prompts, and all samples are then filtered and validated (including senior-physician review) before being used for training and testing. It contains 2,750 dialogues for training and 687 for testing, focusing on hypertension management topics like blood pressure interpretation and medication adjustments. In addition, it contains cases with secondary-hypertension clues and complication/target-organ-damage–related discussions to support long-tail clinical reasoning.

MedDG^32^: A large Chinese medical dialogue dataset with 17,000 doctor-patient dialogues on 12 common internal diseases. About 5,000 dialogues related to hypertension or similar internal medicine patterns were selected for training, improving the model’s ability to generate professional, accurate responses.

MIMIC-III^33^: To enhance the model’s clinical background knowledge, we selected 1000 records related to hypertension and cardiovascular diseases from the MIMIC-III ICU medical record database, which contains over 53,000 adult ICU records. These records were translated into Chinese summaries or Q&A dialogues using a controlled vocabulary aligned with standards such as ICD-10, UMLS, and SNOMED CT to maintain consistent medical terminology. After machine translation, bilingual medical professionals reviewed the translations to verify accuracy and consistency, ensuring the integrity and clinical reliability of the dataset.

CMB^34^ : A benchmark evaluating large models’ medical knowledge, reasoning, and consultation abilities in the Chinese medical context. We selected 500 hypertension-related questions from this dataset for performance testing on the model’s understanding and reasoning in clinical settings. By integrating these datasets, we created a comprehensive, structured Chinese dataset covering hypertension management, providing essential data for fine-tuning and evaluation of large language models in chronic disease management.

#### Data preprocessing and standardization

Although the four corpora listed above differ in source, format and writing style, they are all mapped to a unified representation before model training. First, all samples are converted into the input, cot_reasoning, answer JSON schema used by HTN-5M. For multi-turn dialogue datasets (HTN-5M and MedDG), the concatenated patient utterances form the input, the final physician response is used as the answer, and the intermediate diagnostic logic is stored in the cot_reasoning field. For MIMIC-III summaries, we extract the hypertension-related description as the input and the corresponding management recommendations as the answer. For CMB, the original single-turn question–answer pairs are kept and only used for evaluation.

Second, we standardize clinical terminology and linguistic style across datasets. Based on a curated hypertension lexicon aligned with ICD-10, UMLS and Chinese hypertension guidelines, synonymous expressions (e.g. “high blood pressure”, “elevated BP”, “stage 2 hypertension”) are mapped to a canonical form, measurement units (mmHg, $\textrm{kg}/\textrm{m}^2$ are normalized, and obvious spelling or typographical variants are corrected. We also remove template-like greetings, location information and personally identifiable details, and filter out responses that are overly colloquial or noisy. This reduces domain shift between corpora and makes the supervision signal more homogeneous.

Finally, we check the distribution of samples after integration and adopt a simple balancing strategy to mitigate dataset bias. As summarized in Table 3, HTN-5M, MedDG and MIMIC-III contribute 36.4%, 53.0% and 10.6% of the training examples, respectively, while CMB is reserved for testing only. To prevent the model from overfitting to the general multi-disease style of MedDG, mini-batches are constructed using stratified sampling with comparable probabilities for HTN-5M and MedDG samples and a smaller probability for MIMIC-III. In addition, early stopping and hyper-parameter selection are performed on a validation split that is balanced across the three training sources. In this way, the model is exposed to diverse clinical expressions but no single dataset dominates training, which improves the robustness and generalization ability of ChatHTN on unseen hypertension consultation questions. This multi-source integration and stratified sampling strategy helps reduce the risk of inheriting a single generator’s stylistic biases and mitigates the potential homogeneity associated with purely synthetic supervision.

### Baseline

BenTsao: Based on the LLaMA architecture and LoRA technology for parameter-efficient fine-tuning, this model integrates a knowledge base to improve training and inference. It shows significant improvements in accuracy and reliability, making it suitable for high-quality text generation in complex Q&A tasks.

GPT-3.5-turbo^35^: Optimized from GPT-3, this model improves dialogue interaction, instruction-following, and contextual reasoning by incorporating more training data and architectural enhancements. It excels in multi-turn Q&A and complex contextual tasks.

HuaTuoGPT: By combining ChatGPT fine-tuning data and real-world medical data, this model enhances performance in medical consultation and diagnostic advice. It offers higher accuracy and personalized health suggestions, handling complex medical issues effectively.

DoctorGLM: Built on Tsinghua University’s open-source ChatGLM-6B, this model was fine-tuned with Chinese medical dialogue data, showing strong potential in medical Q&A by generating professional, coherent responses.

ChatGLM3-6B: Developed by Tsinghua University KEG Lab and Zhipu AI, this open-source bilingual model is the third generation of the ChatGLM series. With a self-developed GLM architecture, it provides improved language understanding and generation, optimized for performance and deployment.

DeepSeek-LLM-67B-base^36^: With 67 billion parameters, this model from DeepSeek uses a Transformer Decoder-only architecture. Trained on diverse data, including medical, scientific, and multilingual texts, it excels in domains like medicine, law, and programming. In this paper, the original parameters of the model were used.

### Evaluation metrics

We evaluated the model using BLEU-4^37^, ROUGE-L^38^, METEOR^39^ for language generation, and Accuracy and CMB score for structured tasks like treatment recommendations.

*BLEU-4* measures n-gram precision (up to 4-grams) between generated and reference sentences:

8 $$\begin{aligned} \text {BLEU-4} = \text {BP} \cdot \exp \left( \sum _{n=1}^4 w_n \log p_n \right) , \end{aligned}$$

where $p_n$ is the modified n-gram precision, $w_n$ are uniform weights (e.g. $w_n = \frac{1}{4}$), and $\text {BP}$ is the brevity penalty:

9 $$\begin{aligned} \text {BP} = {\left\{ \begin{array}{ll} 1 & \text {if } c > r \\ e^{(1 - \frac{r}{c})} & \text {if } c \le r \end{array}\right. } \end{aligned}$$

Here, *c* is the candidate length and *r* is the reference length.

*ROUGE-L* evaluates the longest common subsequence (LCS) between generated and reference responses:

10 $$\begin{aligned} \text {ROUGE-L} = \frac{(1 + \beta ^2) \cdot R_{\text {LCS}} \cdot P_{\text {LCS}}}{R_{\text {LCS}} + \beta ^2 \cdot P_{\text {LCS}}}, \end{aligned}$$

where $P_{\text {LCS}} = \frac{LCS}{m}$ and $R_{\text {LCS}} = \frac{LCS}{n}$ are precision and recall, with *m* and *n* being the lengths of candidate and reference texts respectively. $\beta$ is typically set to 1.2.

*METEOR* captures semantic similarity by considering exact, stem, and synonym matches:

11 $$\begin{aligned} & \text {METEOR} = F_{\text {mean}} \cdot (1 - \text {Penalty}) \end{aligned}$$

12 $$\begin{aligned} & F_{\text {mean}} = \frac{10 \cdot P \cdot R}{9 \cdot P + R} \end{aligned}$$

13 $$\begin{aligned} & \text {Penalty} = \gamma \cdot \left( \frac{\textrm{chunks}}{\textrm{matches}} \right) ^\theta . \end{aligned}$$

Typical values are $\gamma = 0.5$, $\theta = 3$. *P* and *R* are unigram precision and recall.

*Accuracy* quantifies overall correctness in classification tasks

14 $$\begin{aligned} \text {Accuracy} = \frac{TP + TN}{TP + TN + FP + FN}. \end{aligned}$$

These metrics offer a clear view of the model’s language generation and decision-making capabilities.

### Problem definition

To objectively compare performance across different model settings, a comprehensive test set was extracted from the HTN-5M dataset, consisting of three difficulty levels: RQ1 (simple), RQ2 (moderate), and RQ3 (complex). Simple questions (RQ1, 30 questions) involve basic symptoms or general inquiries, such as “What is considered high blood pressure?” and “Does hypertension require daily medication?” These questions typically need the identification of a single medical entity and basic medical knowledge.Moderate questions (RQ2, 20 questions) require reasoning across multiple factors, with two or more symptoms, medical history, or medications, such as “I have a headache and a family history; do I need medication?” and “Can I take antihypertensive drugs during pregnancy?”Complex questions (RQ3, 10 questions) involve multi-step reasoning, such as “What should I do if I still feel dizzy and have chest tightness after taking medication?” These questions require comprehensive analysis, time-based reasoning, and understanding symptom progression to generate medically valid and actionable responses. To specifically probe generalization to less frequent but clinically important patterns, we also report performance on the auxiliary etiology-classification and complication-identification tasks, which directly involve secondary causes and target-organ damage. Moreover, we evaluate on an external hypertension-related benchmark (CMB) in addition to the HTN-5M-derived test set to assess robustness under distribution shifts.

### Experimental setup

All the fine-tuning experiments are carried out under the PyTorch 2.2.0 framework, and mainstream distributed training strategies are used to improve training efficiency and stability. The specific hyperparameters are set as follows: the base learning rate is 3e-5, taking into account the convergence speed and stability; the maximum number of input tokens is 5000, and the maximum number of output tokens is 3000, to adapt to the demand for long text input (e.g. medical history, lifestyle) and detailed answers in the hypertension diagnosis scenario; top_p and temperature are set to 0.7 and 0.95, respectively, to balance the diversity and certainty of generated content.

## Experiments and analysis

### Overall performance on hypertension consultation benchmarks

The ChatHTN model integrates a hypertension-specific knowledge graph with multi-task fine-tuning, showing clear advantages over other models as presented in Table 4. The structured knowledge graph strengthens the model’s grasp of medical terminology and relationships among disease entities, enabling more accurate and consistent responses in complex cases that involve reasoning about etiology, symptoms, and interventions. This design helps prevent hallucinations common in general models such as ChatGPT-3.5 and DeepSeek-LLM-67B. Meanwhile, the multi-task fine-tuning enhances entity recognition, etiology classification, and intervention suggestion generation, improving overall reliability. The slightly lower BLEU-4 score of ChatHTN compared with HuaTuoGPT arises from its emphasis on structured reasoning and clinical accuracy rather than linguistic fluency; HuaTuoGPT’s broader training corpus yields smoother expressions but weaker medical logic. Using DeepSeek-LLM-67B’s original parameters provided a robust baseline to verify that the targeted fine-tuning and knowledge-graph integration of ChatHTN yield substantial performance gains in domain-specific medical question answering.

**Table 4:** Performance of different models on various evaluation metrics (%).

Model	BLEU-4	ROUGE-L	METEOR	CMB
ChatGPT-3.5	9.13	19.72	21.45	34.75
HuaTuoGPT	15.67	24.36	26.97	42.50
BenTsao	6.54	16.94	19.52	35.00
DoctorGLM	10.89	21.25	23.46	38.25
ChatGLM3-6B	2.43	9.87	12.53	27.25
DeepSeek-LLM-67B-Base	4.21	12.62	14.79	32.50
ChatHTN	12.78	30.84	29.25	48.75

**Table 5:** Statistical significance testing results of ChatHTN compared with baseline models.

Metric	ChatHTN	Best Baseline	Improvement	*p*-**value**	95% confidence interval
BLEU-4	12.78	10.89 (DoctorGLM)	+1.89	**0.013**	[0.52, 3.07]
ROUGE-L	30.84	24.36 (HuaTuoGPT)	+6.48	0.009	[3.21, 9.42]
METEOR	29.25	26.97 (HuaTuoGPT)	+2.28	0.021	[0.41, 4.17]
CMB	48.75	42.50 (HuaTuoGPT)	+6.25	0.004	[3.12, 9.05]

### Statistical significance analysis

To further evaluate the robustness and reliability of the reported performance improvements, we conducted statistical significance testing across the main evaluation metrics (BLEU-4, ROUGE-L, METEOR, and CMB). A paired t-test was applied between the proposed ChatHTN model and baseline models (HuaTuoGPT, DoctorGLM, and ChatGPT-3.5) on the test sets. The statistical analysis results demonstrate that the performance gains of ChatHTN are statistically significant across all metrics. Specifically, the mean metric values and p-values are summarized in Table 5 . To ensure comprehensive comparison, we also performed a one-way ANOVA across all evaluated models, which yielded F(5, 24) = 8.42, p < 0.001, confirming significant performance differences among models. These results confirm that the observed improvements of ChatHTN are not due to random fluctuations but are statistically reliable.

### Effect of loss weight configuration

To make the proposed multi-task fine-tuning framework more transparent and reproducible, we further analyze how the choice of loss weights for the four subtasks (H-MER, H-EC, H-CI, and H-ISG) affects the model’s performance. Specifically, we select several representative weight combinations from the grid search described in Sect. "Multi-task knowledge fine-tuning strategy" and report the validation results in Table 6. For each configuration, we train ChatHTN on the training set using the corresponding $(\lambda _{\text {MER}}, \lambda _{\text {EC}}, \lambda _{\text {CI}}, \lambda _{\text {ISG}})$ and evaluate BLEU-4, ROUGE-L, METEOR, and CMB on the held-out validation set.

**Table 6:** Validation performance under different loss weight configurations for the four subtasks.

Config	$(\lambda _{\text {MER}}, \lambda _{\text {EC}}, \lambda _{\text {CI}}, \lambda _{\text {ISG}})$	BLEU-4	ROUGE-L	METEOR	CMB
Config A (equal)	(1.0, 1.0, 1.0, 1.0)	11.02	28.75	27.10	45.20
Config B (ours)	(0.8, 0.6, 0.6, 1.0)	12.78	30.84	29.25	48.75
Config C (ISG$\uparrow$)	(0.5, 0.5, 0.5, 1.2)	11.40	29.35	27.60	46.10
Config D (MER$\uparrow$)	(1.2, 0.6, 0.6, 1.0)	10.85	28.20	26.90	45.05

The selected configuration (Config B) is used in all subsequent experiments.

**Fig. 5:**
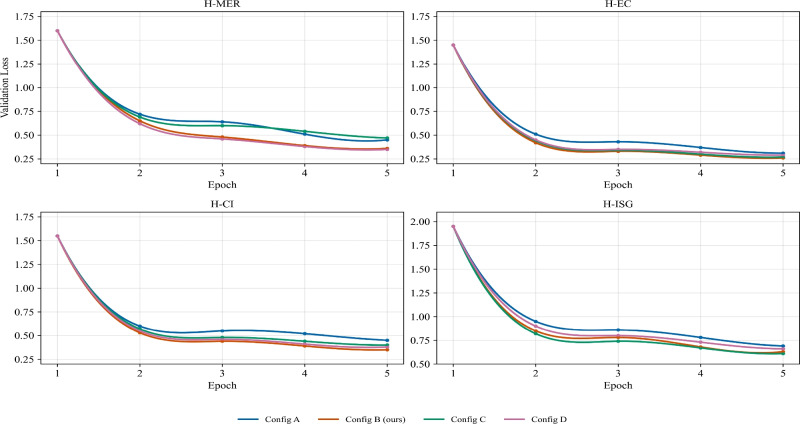
Loss values for each module under different configurations.

From Table 6, we observe that the equal-weight configuration (Config A) already provides a strong baseline. However, Config B, which slightly up-weights the main intervention suggestion task (H-ISG) while assigning moderate weights to the auxiliary subtasks, achieves the best overall validation performance, improving BLEU-4, ROUGE-L, METEOR, and CMB by approximately 1.76, 2.09, 2.15, and 3.55 points, respectively, compared with Config A. In contrast, excessively emphasizing H-ISG (Config C) or over-weighting the entity recognition loss (Config D) leads to small decreases in BLEU-4 and CMB, indicating that an imbalanced focus on a single subtask can harm the global quality of consultation responses. These results support the use of the weight setting in Config B, which strikes a good balance between the four subtasks and is therefore adopted as the default configuration in our main experiments.Key loss values are shown in Fig. 5.

### Performance across question difficulty levels (RQ1–RQ3)

**Fig. 6:**
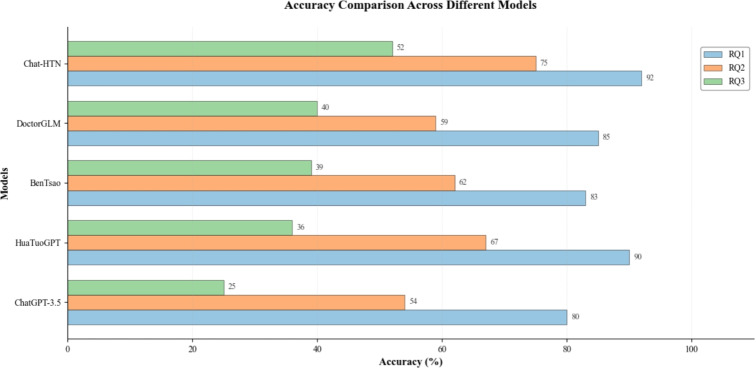
Model accuracy on three QA tasks.

As shown in Fig. 6, the ChatHTN model achieved significantly higher accuracy across all three question-answering tasks (RQ1: symptom identification, RQ2: personalized treatment suggestions, RQ3: contextual reasoning), with accuracies of 92%, 75%, and 52%, respectively. Compared with other mainstream models, ChatHTN maintained clear advantages in both reasoning depth and medical consistency. However, its accuracy in RQ3 declined due to the increased complexity of multi-turn reasoning and evolving symptom descriptions. In such cases, the model occasionally failed to establish causal links between symptoms, treatments, and complications, leading to recommendations that diverged from clinical guidelines. For example, when handling continued dizziness after medication, it sometimes overlooked the interaction between the drug and the patient’s condition.

**Fig. 7:**
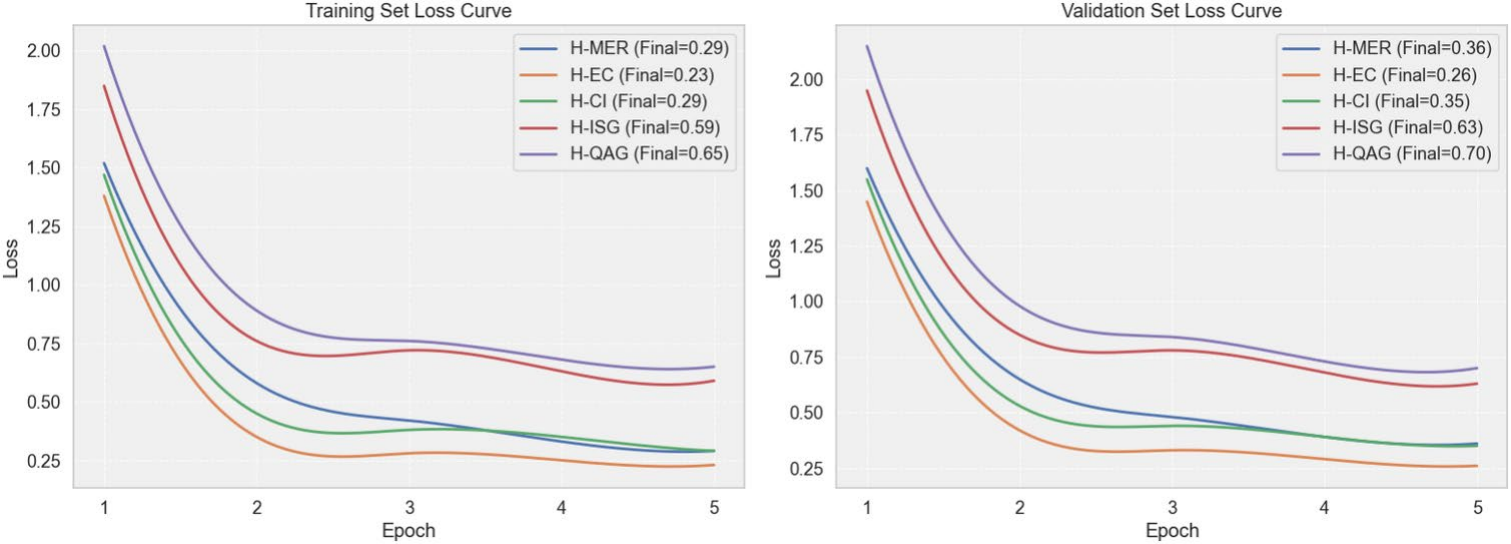
Loss and Acc curves.

As illustrated in Fig. 7, the training and validation loss curves further confirm the stability and convergence of ChatHTN during optimization, highlighting its robustness across tasks. The superior performance of the ChatHTN model across all tasks is largely due to its integration of a structured knowledge graph and multi-task collaborative mechanism. This enables the model not only to accurately identify medical entities but also to effectively combine multi-dimensional information such as etiology, symptoms, and interventions when addressing complex questions, such as “What should I do if I still feel dizzy with blurred vision after taking medication?” The comparison with other models shows that while general-purpose models excel in language capabilities, they lack the support of specialized knowledge. On the other hand, models fine-tuned in the medical domain may have relevant background but struggle to leverage structured reasoning effectively.

**Fig. 8:**
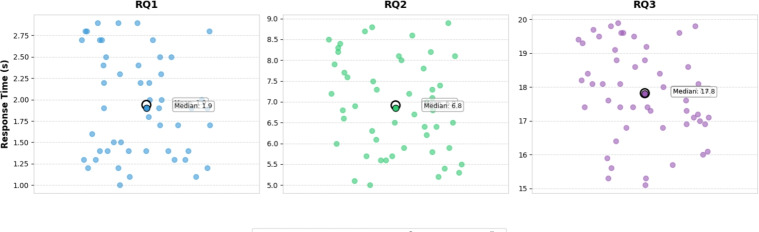
System response time across three question categories.

As shown in Fig. 8, to improve the inference speed of the model, we converted the model format to ONNX during deployment and optimized it using TensorRT. The response times for different levels of question complexity (RQ1, RQ2, RQ3) exhibit clear stratification. For RQ1, the response times are primarily between 1 and 3 seconds, indicating that the system can quickly handle simple, structured queries. For RQ2, which involves more complex reasoning, the response times range from 4 to 8 seconds, reflecting the higher computational demands. In contrast, for RQ3, which involves multi-turn reasoning and longer text processing, the response times are mostly concentrated between 10 and 16 seconds, with the average being higher than the other tasks. The increased response times are due to the challenges posed by more complex tasks, which require deeper reasoning and longer computation cycles. Overall, as task difficulty increases, response times also increase, with greater fluctuations observed in hig.

### Ablation study of knowledge graph and multi-task components

In the Table 7, R-KG represents the interaction without the knowledge graph, and R-Finetuning indicates the removal of multi-task knowledge fine-tuning, using only the original parameters of ChatGLM3-6B. From the experimental data, the ChatHTN model achieved the best performance across all task types, with an accuracy of 92% in RQ1, 75% in RQ2, and 52% in RQ3, resulting in an average accuracy of 73%, significantly outperforming other models. In the R-KG model, after removing the knowledge graph interaction, the accuracy in RQ1 remained high at 91%, but dropped to 63% and 43% in RQ2 and RQ3, respectively, with an average accuracy of 65.7%. This shows that the knowledge graph plays a crucial role in etiology analysis and complex consultation reasoning. The R-Finetuning model further removed multi-task knowledge fine-tuning and relied solely on the original model parameters. Its performance in RQ2 and RQ3 significantly decreased to 54% and 32%, respectively, indicating the importance of the multi-task mechanism in enhancing the model’s logical reasoning capabilities. In contrast, general-purpose models such as ChatGLM3-6B and Deepseek-LLM-67B-base performed poorly overall, especially in RQ3 multi-turn reasoning tasks, where their accuracy was only 20% and 27%, with average accuracies of 48.3% and 54%, respectively. This suggests that they lack effective knowledge support and task-specific design when addressing domain-specific problems. Overall, the ChatHTN model, by integrating a structured medical knowledge graph and multi-task collaborative mechanism, significantly enhances the model’s capabilities in medical entity recognition, intervention suggestion generation, and contextual understanding, demonstrating strong adaptability and generalization ability in hypertension consultation scenarios.

**Table 7:** Ablation study results (%).

Model	RQ1	RQ2	RQ3	AVG
R-KG	91	63	43	65.7
R-Finetuning	80	54	32	55.3
ChatGLM3-6B	78	47	20	48.3
DeepSeek-LLM-67B-Base	85	50	27	54.0
ChatHTN	92	75	52	73.0

### Preliminary cross-disease transfer experiment on diabetes

To further investigate whether the proposed ChatHTN framework can be adapted to other chronic diseases, we conducted a preliminary cross-disease transfer learning experiment on diabetes-related question answering. Although all main experiments in this work were performed on hypertension, the model architecture is designed to be disease-agnostic and theoretically applicable to other chronic conditions. Therefore, this supplementary experiment aims to provide initial empirical evidence regarding the potential transferability of ChatHTN beyond the hypertension domain.

*Construction of the diabetes dataset.* Following the same data structuring process used in HTN-5M, we extracted diabetes-related doctor–patient dialogues and single-turn question–answer pairs from the MedDG corpus, which originally covers 12 common internal diseases. After removing incomplete conversations, duplicates, and samples lacking specific clinical recommendations, a diabetes subset (denoted as *DIA-QA*) containing 800 training instances and 200 test instances was obtained. All records were reformatted into the unified schema of {*input*, *cot_reasoning*, *answer*}, and the same terminology normalization process described in Sect. "Composition of the dataset" was applied to ensure consistency.

*Compared methods.* We evaluated the following four settings:

1. *ChatGLM3-6B (zero-shot):* the original ChatGLM3-6B model is directly evaluated on DIA-QA without any fine-tuning;
2. *ChatHTN (zero-shot):* the hypertension-specialized model proposed in this paper is directly applied to DIA-QA;
3. *ChatGLM3-6B + Diabetes FT:* ChatGLM3-6B is fine-tuned on the DIA-QA training set using the standard question answering objective;
4. *ChatHTN + Diabetes FT (ours):* initialized from the hypertension-trained ChatHTN model and further fine-tuned on DIA-QA.

Hyperparameters were kept exactly the same for settings 3 and 4, including learning rate, batch size and the number of training epochs, to ensure a fair comparison.

**Table 8:** Preliminary transfer learning results on the diabetes question-answering dataset (DIA-QA).

Model	BLEU-4	ROUGE-L	METEOR	CMB
ChatGLM3-6B (zero-shot)	3.21	10.42	13.58	29.10
ChatHTN (zero-shot)	4.05	13.27	15.02	31.85
ChatGLM3-6B + Diabetes FT	8.76	22.48	23.71	40.22
ChatHTN + Diabetes FT (ours)	10.34	25.96	25.89	43.47

The results are presented in Table 8. It can be observed that: (i) in the zero-shot scenario, ChatHTN already performs slightly better than ChatGLM3-6B, suggesting that part of the reasoning strategy learned from hypertension consultations transfers to diabetes even without additional tuning; and (ii) after fine-tuning, *ChatHTN + Diabetes FT* consistently outperforms *ChatGLM3-6B + Diabetes FT* across all four metrics, with the most notable improvement observed in ROUGE-L and CMB, indicating better retention of clinically relevant details and guideline-aligned intervention suggestions. These findings provide preliminary but concrete evidence that the proposed knowledge-graph–enhanced multi-task framework captures reusable consultation and reasoning patterns that can be adapted to other chronic diseases with relatively limited additional data. Nevertheless, due to the small size of DIA-QA and the focus on a single secondary disease, a comprehensive cross-disease evaluation on larger diabetes or multi-morbidity datasets will be required in future work to more rigorously verify the framework’s generalization capability.

*Scalability and adaptation costs.* While the preliminary transfer results are encouraging, we must acknowledge the significant resource overhead required to replicate the full ChatHTN pipeline for new diseases. The model’s superior performance is deeply rooted in the domain-specific Hypertension Knowledge Graph (HTN-KG) and the expert-validated CoT dataset (HTN-5M). As detailed in Sect. "Method", constructing these resources involved rigorous data cleaning, schema definition, and multi-round physician reviews, which constitutes a substantial “cold-start” cost. Extending this framework to other complex conditions (e.g. Diabetes, COPD) or co-morbidities would necessitate similar domain-specific engineering efforts, potentially limiting rapid scalability. Therefore, the “transferability” demonstrated here refers to the model architecture and learning strategy, while the data pipeline remains a resource-intensive bottleneck. Future research must explore automated KG construction and low-resource CoT generation techniques to mitigate these adaptation costs and facilitate broader deployment.

### Discussion on robustness to real-world conversational noise

Real-world clinical consultations are often more colloquial, fragmented, and ambiguous than the standardized datasets used in training. While our current evaluation focuses on structured data, the ChatHTN framework incorporates specific design features intended to mitigate the impact of such conversational noise:

- Attentional Filtering via Multi-task Learning: The Hypertension Medical Entity Recognition (H-MER) subtask explicitly trains the model to identify and extract key medical terms (e.g. symptoms, medication names) from the input text. This mechanism encourages the model to focus its attention weights on clinically relevant information while down-weighting irrelevant colloquial fillers or fragmented phrasing. Effectively, H-MER acts as a “noise filter” that extracts the medical signal from the linguistic noise.
- Semantics Grounding via Knowledge Graph: The integration of the Hypertension Knowledge Graph (HTN-KG) provides a grounding mechanism for ambiguous terms. By projecting input tokens into the KG embedding space, colloquial expressions that map to valid medical entities are reinforced, while ambiguous or irrelevant terms that lack graph correspondence have less influence on the reasoning path.
- Safeguard in Deployment: Finally, as detailed in Sect. "Clinical safety and ethical considerations", ChatHTN is designed to operate within a Human-in-the-Loop workflow. In cases where the input is highly ambiguous or the “messiness” exceeds the model’s resolution capability, the system is designed to flag low-confidence predictions for physician review, ensuring that linguistic ambiguity does not lead to clinical errors.

## Conclusion

This study proposes ChatHTN, a model that integrates a hypertension knowledge graph and multi-task fine-tuning to improve medical understanding and personalized interventions for hypertension. We developed the HTN-5M dataset and combined TransE-based knowledge-graph embeddings with multi-task learning, achieving significant improvements in BLEU, ROUGE, and accuracy, particularly in complex reasoning tasks. Ablation studies further confirm the crucial role of the knowledge graph and multi-task mechanism in improving performance. Although the main experiments in this work focus on hypertension, a preliminary transfer learning experiment on diabetes question answering (Sect. "Ablation study of knowledge graph and multi-taskcomponents") shows that ChatHTN adapted with a small amount of diabetes data outperforms a ChatGLM3-6B baseline trained on the same dataset, providing concrete evidence for the feasibility of extending this framework to other chronic diseases. Future research will focus on two key directions. First, we aim to further generalize and validate ChatHTN on larger-scale diabetes and multi-morbidity datasets to strictly assess its clinical robustness. Second, to address the scalability challenges highlighted in our discussion, we plan to investigate automated knowledge extraction and weak-supervision methods. This will help reduce the reliance on expensive manual expert annotation when adapting the system to new medical domains. Additionally, a dynamic updating mechanism could be introduced to improve the timeliness and accuracy of the knowledge graph, and multi-modal information, including electronic medical records and medical images, could be incorporated for more comprehensive medical context modeling. Furthermore, addressing the challenge of knowledge obsolescence is critical. We plan to transition from the current periodic update mechanism to a continuous learning framework, allowing the Knowledge Graph to automatically flag and assimilate new findings from daily-updated medical publication databases (e.g. PubMed), thereby reducing the latency of knowledge maintenance. Finally, deploying the system in clinical environments, combined with doctor feedback, could enable continuous model optimization, facilitating the integration and adoption of large language models in chronic disease management.

## Clinical safety and ethical considerations

Although the ChatHTN system demonstrates strong reasoning and consultation capabilities, its deployment in real clinical environments requires strict safety and ethical validation. To move from an experimental setting to real-world clinical use, we define a phased implementation plan, clarify the division of responsibilities among stakeholders, and design a safety-aware decision-making workflow that keeps licensed physicians firmly “in the loop”.

### Phased clinical validation and collaboration with hospitals

In the first phase, ChatHTN will be evaluated in an offline setting using retrospective consultation records provided by collaborating hospitals. Model outputs (including reasoning chains and intervention suggestions) will be compared against expert annotations and guideline-based recommendations to quantify accuracy, consistency, and potential safety risks. In the second phase, a small-scale prospective pilot will be conducted in partner hospitals under ethics committee approval. In this stage, the system will operate as an internal decision-support tool: physicians can view AI-generated suggestions alongside the patient’s clinical information, but only physician-confirmed decisions will be recorded in the electronic medical record. In the third phase, if safety and effectiveness thresholds are met, the deployment scope can be gradually expanded to more departments and institutions, with continuous monitoring of performance and adverse events. Throughout all phases, formal collaboration agreements with hospitals will define data sharing procedures, feedback mechanisms, and criteria for suspending or updating the system if safety concerns arise.

### Roles and responsibilities

Clear assignment of responsibilities is essential for safe deployment. The research team is responsible for model development, version control, performance monitoring, and technical documentation. Collaborating hospitals, through their clinical departments, are responsible for clinical validation, feedback on model behavior, and final medical decision-making. Hospital ethics committees or institutional review boards (IRBs) are responsible for reviewing and approving study protocols, ensuring that the use of ChatHTN complies with ethical principles and protects patient rights. Data protection and compliance officers are responsible for supervising data handling, including de-identification, secure storage, and access control, in accordance with applicable medical data protection regulations and hospital policies.

### Human-in-the-loop decision-making workflow

To ensure that ChatHTN functions strictly as a decision-support system rather than an autonomous diagnostic agent, a human-in-the-loop workflow will be adopted. First, the patient’s structured information and free-text complaints are input into the system. Second, ChatHTN generates a preliminary reasoning chain and consultation recommendation. Third, the system automatically flags high-risk cases (e.g. suspected hypertensive emergencies, severe comorbidities, or medication contraindications) based on predefined clinical rules and guideline-based thresholds. Fourth, all AI-generated outputs are presented on a clinician dashboard, where the responsible physician can *accept*, *modify*, or *reject* the suggestions. Only physician-approved recommendations are communicated to the patient or written into the medical record, and any modifications are logged to support audit and further model improvement. This workflow ensures that clinical responsibility remains with licensed physicians at all times.

### Regulatory and ethical compliance framework

The deployment and evaluation of ChatHTN will be conducted under the oversight of hospital ethics committees and in accordance with established medical ethical principles (e.g. the Declaration of Helsinki) and good clinical practice guidelines. Before any prospective clinical use, protocols involving ChatHTN must undergo institutional ethics review, and informed consent procedures will be implemented where required. From a data protection perspective, the system will comply with relevant national regulations on personal information protection and electronic health records, including strict de-identification of training data, access control, and audit logs for all model queries and outputs. In addition, any future updates of the model or knowledge graph will be versioned and, where necessary, re-evaluated through the same validation pipeline to avoid unmonitored changes in behavior. By combining a phased validation schedule, clearly defined responsibilities, a transparent decision workflow, and alignment with clinical and data protection regulations, ChatHTN is positioned to be integrated into chronic disease management in a controlled and ethically robust manner.

## Data Availability

The processed data is available in the https://osf.io/ye9g6/?view_only=00d9593f7a1e44969bd061b99034b9ea; 10.17605/OSF.IO/YE9G6.
